# Secretion of *Salmonella* Pathogenicity Island 1-Encoded Type III Secretion System Effectors by Outer Membrane Vesicles in *Salmonella enterica* Serovar Typhimurium

**DOI:** 10.3389/fmicb.2018.02810

**Published:** 2018-11-23

**Authors:** Seul I Kim, Seongok Kim, Eunsuk Kim, Seo Yeon Hwang, Hyunjin Yoon

**Affiliations:** ^1^Department of Molecular Science and Technology, Ajou University, Suwon, South Korea; ^2^Department of Applied Chemistry and Biological Engineering, Ajou University, Suwon, South Korea

**Keywords:** *Salmonella*, outer membrane vesicles (OMVs), *Salmonella* pathogenicity island 1 (SPI-1), type III secretion system (T3SS), virulence, effector

## Abstract

Outer membrane vesicles (OMVs) are spherical membranous structures released by Gram-negative bacteria. Several bacterial pathogens utilize OMVs as vehicles for the delivery of virulence factors into host cells. Results of our previous study on proteomic analysis revealed that OMVs isolated from *Salmonella* *enterica* serovar Typhimurium had virulence effectors that are known to be translocated by *Salmonella* pathogenicity island 1 (SPI-1)-encoded type III secretion system (T3SS1) into the host cell. In the present study, immunoblot analysis confirmed the secretion of the six T3SS1 effector proteins, namely SipB and SipC (translocators of T3SS1), and SipA, SopA, SopB, and SopE2 (effectors translocated by T3SS1), by OMVs. Results of proteinase K treatment revealed the localization of these T3SS1 effector proteins on the outer surface of OMVs. SipC and SopE2 were secreted by OMVs independent of the three secretion systems T3SS1, T3SS2, and flagella, signifying OMVs to be an alternative delivery system to T3SSs. T3SS1 effectors SipA, SipC, and SopE2 were internalized into the cytoplasm of the host cell by OMVs independent of cellular *Salmonella–*host cell contact. In epithelial cells, addition of OMVs harboring T3SS1 effectors stimulated the production of F-actin, thereby complementing the attenuated invasion of Δ*sopE2* into host cells. These results suggest that *S.* Typhimurium might exploit OMVs as a long-distance vehicle to deliver T3SS1 effectors into the cytoplasm of the host cell independent of bacteria–host cell interaction.

## Introduction

*Salmonella enterica* serovar Typhimurium, a subspecies of *Salmonella enterica*, is a Gram-negative pathogenic bacterium. Salmonellae infect a wide range of animals including humans and cause various diseases, ranging from gastroenteritis to typhoid fever (Chimalizeni et al., 2010). During the course of its evolution into a pathogen, *Salmonella* has acquired several genetic elements that enable it to compromise host immune responses and to persist within host cells (Winter et al., 2010; Thiennimitr et al., 2012). *Salmonella* pathogenicity island 1 (SPI-1) and SPI-2 are examples of the most important *Salmonella* genetic determinants involved in virulence. They encode structural components that form sophisticated molecular machinery called type III secretion systems (T3SSs) and multiple cognate virulence factors that are secreted through these systems. The SPI-1 T3SS (T3SS1) is primarily associated with the early stage of infection where it translocates T3SS1 effectors across the host cell membrane for bacterial invasion of intestinal epithelial cells and stimulation of intestinal inflammation (Galan, 2001; McGhie et al., 2009). The SPI-2 T3SS (T3SS2) translocates multiple effectors to the host cell cytosol that alter host cellular functions, thereby promoting intracellular bacterial survival and replication (Galan, 2001; McGhie et al., 2009). Due to the structural similarity between flagellum and T3SS, *Salmonella* exploits its flagellar apparatus not only for locomotion but also for translocation of virulence factors into eukaryotic host cells (Young et al., 1999; Young and Young, 2002; Diepold and Armitage, 2015). Although *Salmonella* has other secretion mechanisms, including an autonomous secretion system ZirT/ZirS (Ehrbar et al., 2003) and a type VI secretion system (Bingle et al., 2008), the two T3SSs and the flagella are considered as the primary secretion machineries associated with the virulence of *Salmonella* (Diepold and Armitage, 2015).

Gram-negative bacteria ubiquitously secrete outer membrane vesicles (OMVs), which are nanoscale proteoliposomes ranging in size between 20 and 250 nm in diameter (Kuehn and Kesty, 2005; Mashburn and Whiteley, 2005). Biochemical and proteomic analyses reveal that OMVs consist of a wide range of molecules present in the outer membrane, periplasm and inner membrane; and in the cytoplasm, including soluble proteins, integral membrane proteins, lipoproteins, glycolipids, lipopolysaccharide (LPS), toxins, and DNA (Lee et al., 2007; Kulp and Kuehn, 2010; Choi et al., 2011). Recent studies have reported an association between OMVs and bacterial physiology and pathogenicity in many pathogens, including *Escherichia coli, Helicobacter pylori*, and *Pseudomonas aeruginosa* (Schwechheimer and Kuehn, 2015). OMVs are a means by which Gram-negative bacteria dispose of intracellular garbage such as misfolded proteins and abnormal envelope fragments (LPS and peptidoglycan fragments) that accumulate under stressful conditions (Maredia et al., 2012; Macdonald and Kuehn, 2013; Schwechheimer and Kuehn, 2015). Pathogenic bacteria such as *E. coli* and *Vibrio cholera* use OMVs as decoys to confront and dilute antibiotics (Manning and Kuehn, 2011; Duperthuy et al., 2013), while *P. aeruginosa* and *Staphylococcus aureus* use OMVs to deliver antibiotic degrading enzymes, to resist antibiotic treatment (Ciofu et al., 2000; Lee et al., 2013). In addition, OMVs carry and disseminate enzymes that break down macromolecules to accessible carbon and nitrogen compounds (Evans et al., 2012; Biller et al., 2014; Elhenawy et al., 2014) and carry iron and zinc acquisition systems that scavenge these metals from the environment for bacterial growth (Lappann et al., 2013; Veith et al., 2014). OMVs contribute to bacterial virulence by delivering virulence factors and by modulating host immune responses (Balsalobre et al., 2006; Kaparakis-Liaskos and Ferrero, 2015). *P. aeruginosa* utilizes OMVs to translocate cystic fibrosis transmembrane conductance regulator (CFTR)-inhibitory factor (Cif) into host cells, which then dampens CD8^+^ T cells-mediated pathogen recognition by stimulating ubiquitination of an ATP-binding cassette (ABC) transporter called transporter associated with antigen processing (TAP)1 (Bomberger et al., 2014). *V. cholera* OMVs transport *V. cholera* cytolysin (VCC), a pore-forming toxin, to trigger an autophagy response in host cells (Elluri et al., 2014). Enterotoxigenic *E. coli* release OMVs to deliver active heat-labile enterotoxin (LT) to the host cell cytosol by endocytosis through cholesterol-rich lipid rafts (Kesty et al., 2004).

For understanding the regulation of virulence in *Salmonella*, it is necessary to identify cargo proteins secreted by OMVs under conditions resembling infection in the host. In our previous study, we performed proteomic profiling of *Salmonella* OMVs that were isolated under two different growth conditions: using Luria-Bertani (LB) medium to represent the standard laboratory conditions and an acidic minimal medium (AMM) to mimic the intracellular environment (Bai et al., 2014). Our results revealed that OMVs contained a variety of T3SS effector proteins known to be translocated into host cells by specialized cognate T3SSs, suggesting the possibility of exploiting OMVs as an alternative vehicle for transporting T3SS effectors in *Salmonella*. In the present study, we examined the possibility and the role of OMVs in translocating T3SS effectors to regulate virulence in *Salmonella*.

## Materials and Methods

### Bacterial Strains and Plasmids

*Salmonella enterica* serovar Typhimurium 14028 (ATCC 14028) was used as the parent strain in the present study. All deletion and tagged strains were constructed using the phage lambda (λ) Red recombination system or P22 HT105/1 int-201-mediated transduction method as described previously (Wing, 1968; Chan et al., 1972;Datsenko and Wanner, 2000). The phage λ Red recombination system was used for the homologous recombination processes, wherein the kanamycinresistance (*kan*) cassette of pKD13 or pKD13-2HA was amplified by polymerase chain reaction (PCR) with 40-nucleotide (nt) flanking sequences homologous to target genes at both termini as described previously (Yoon et al., 2011). Subsequent PCR products were introduced into recipient cells harboring pKD46 to replace the target gene with a *kan* cassette or to insert the HA-coding fragment (Yoon et al., 2011). Antibiotic marker genes were subsequently removed by flip recombinase from pCP20 to make non-polar in-frame deletions (Yoon et al., 2011). Alternatively, P22 HT105/1 int-201-mediated transduction was used to transfer genetic alleles marked with antibiotic resistance cassettes between bacterial strains. Strains and plasmids used in the study are listed in Supplementary Table S1. All primers used for the construction of bacterial strains are listed in Supplementary Table S2.

### Bacterial Growth Conditions

*Salmonella* strains were cultured in LB broth to mimic the *in vivo* conditions required for inducing *Salmonella* T3SS1 genes, and for the translation and secretion of their products (Yoon et al., 2009; Ibarra et al., 2010; Bai et al., 2014). *Salmonella* cells were pre-cultured overnight in LB broth at 37°C and then diluted 1:100 in fresh LB broth for further growth at 37°C. The following concentrations of appropriate antibiotics were used when required for selection: ampicillin, 50 μg/mL; chloramphenicol, 35 μg/mL; and kanamycin, 50 μg/mL.

### OMVs Purification

*Salmonella* cells cultured in LB broth (500 mL or 1 L) were centrifuged at 10,000 ×*g* for 5 min. The cell-free supernatant was then filtered through a polyvinylidene difluoride (PVDF) filter (0.45-μm-pore-size, Millipore, United States) to remove the remaining bacteria. The filtrate was concentrated by ultrafiltration in a stirred diffusion cell (Thermo Fisher Scientific, United States) using ultrafiltration disks with a molecular weight cutoff (MWCO) of 100 kDa (Thermo Fisher Scientific). The retentate was filtered through a 0.45-μm-pore-sized PVDF membrane to filter remaining bacteria and the filtrate was subjected to ultracentrifugation at 210,100 × *g* for 3 h at 4°C using a SW 41Ti rotor (Optima XE-90, Beckman Coulter, United States). The supernatant was carefully removed and the pellet containing vesicles was suspended in 800 μL of Dulbecco’s phosphate-buffered saline (DPBS). Cell-free preparation of vesicle fractions was examined by spreading a portion of vesicles on LB plates. The purity of OMVs was examined by TEM (transmission electron microscopy) (Supplementary Figure S1). OMVs were quantified using a bicinchoninic acid (BCA) protein assay kit (Thermo Fisher Scientific).

### Immunoblot Analysis

*Salmonella* cells producing HA-tagged T3SS1 effectors were harvested by centrifugation and suspended in 1 × Laemmli sample buffer. OMVs, harvested as described in the above section were precipitated with 20% trichloroacetic acid (TCA) and dissolved in 1 × Laemmli sample buffer. Proteins were separated by sodium dodecyl sulfate polyacrylamide gel electrophoresis (SDS-PAGE) using 10% or 12% polyacrylamide gels and the separated proteins were transferred to PVDF membrane (Bio-Rad, United States). The membranes were blocked with 5% skim milk to prevent non-specific protein binding. T3SS effectors tagged with HA and DnaK were identified using anti-HA (1:10,000 dilution; Sigma, United States) and anti-DnaK (1:10,000 dilution; Enzo Life Sciences, United States) primary antibodies, respectively, in combination with a secondary antibody conjugated with horseradish peroxidase (1:700 dilution; Santa Cruz Biotechnology, United States). OmpA was identified using mouse anti-OmpA antiserum as a primary antibody in a 1:10,000 dilution. The anti-OmpA antiserum was kindly provided by Dr. Ching-Hao Teng (National Cheng Kung University, Taiwan). Immunoblots were developed using West-ZOL Plus western blot detection system (iNtRON Biotechnology, South Korea) according to the manufacturer’s instructions. Images were acquired with ChemiDoc MP System (Bio-Rad).

### Proteinase Accessibility Assay

Purified OMVs were treated with 0.5 μg/mL of proteinase K (Sigma) at 37°C for 30 min and subsequently subjected to overnight TCA precipitation at 4°C. The presence of proteins of interest in proteinase K-treated OMVs was determined using immunoblot analysis as described previously (Muralinath et al., 2011).

### Immunofluorescence Microscopy Analysis

HeLa cells were maintained in Dulbecco’s modified Eagle’s medium (DMEM) supplemented with 4.5 g/L glucose (Thermo Fisher Scientific) and 10% fetal bovine serum (FBS) (Gibco, United States). Cells were washed with phosphate buffered saline (PBS) (Thermo Fisher Scientific), detached with TrypLE express (Thermo Fisher Scientific), and seeded on 12 mm glass cover slips (SPL, South Korea) in 24-well culture plates (SPL) at a density of 2 × 10^5^ cells/well (Kalghatgi et al., 2011). Cells were incubated in DMEM containing 4.5 g/L glucose and 10% FBS at 37°C and 5% CO_2_ for 12 h. To locate T3SS1 effectors of OMVs in HeLa cells, 100 μL of OMVs fraction (corresponding to approximately 50 μg of protein) was administered to host cells and incubated at 37°C and 5% CO_2_ for 2 h. Cells were fixed in 4% formaldehyde for 10 min, washed with PBS, and permeabilized with blocking buffer [PBS supplemented with 0.1% saponin, 1% bovine serum albumin (BSA), and 10% goat serum] for 1 h. Due to reversible permeabilization of cells by saponin, saponin was added at 0.1% in following antibody treatments when required. OMVs were labeled with rabbit anti-*Salmonella* antibody (1:1,000 dilution; Abcam, United Kingdom) and Alexa Fluor 405-conjugated goat anti-rabbit IgG (1:500 dilution; Abcam) or mouse anti-*Salmonella* Typhimurium LPS antibody (1:500 dilution; Abcam) and Alexa Fluor 350-conjugated goat anti-mouse IgG (1:500 dilution; Thermo Fisher Scientific). HA was detected using mouse anti-HA antibody (1:200 dilution; Abcam) and Alexa Fluor 488-conjugated goat anti-mouse IgG (1:500 dilution; Abcam) or chicken anti-HA antibody (1:4,000 dilution; Abcam) and Alexa Fluor 488-conjugated goat anti-chicken IgY (1:1,000 dilution; Abcam). Plasma membranes were stained with Cell Mask Deep Red (Thermo Fisher Scientific). LAMP1 protein was indentified using rabbit anti-LAMP1 antibody (1:4,000 dilution; Abcam) and Alexa Fluor 647-conjugated donkey anti-rabbit IgG (1:1,000 dilution; Abcam). The coverslips were washed with PBS, mounted with BrightMount (Abcam), and analyzed using a DMi8 microscope (Leica, Germany).

In CCF4-AM cleavage assay, HeLa cells were seeded in Lab-Tek II chamber coverglass slides (Thermo Fisher Scientific) and treated with OMVs harboring SipC-Bla or *Salmonella* strains producing SipC-Bla. CCF4 cleavage by Bla activity was examined using LiveBlazer^TM^ FRET-B/G Kit (Thermo Fisher Scientific) by following the instructions provided by the manufacturer and the cleaved CCF4 was analyzed using a DMi8 microscope with emission filter sets at 528 nm (green fluorescence) and 457 nm (blue fluorescence). *Salmonella* cells producing mCherry fluorescence were detected using excitation and emission wavelengths at 560 nm and 610 nm, respectively.

To understand the interaction between OMVs and HeLa cells, OMVs were fluorescently labeled with octadecyl rhodamine B chloride (R18; Thermo Fisher Scientific) as described previously (Fabrega et al., 2016). OMVs were incubated with R18 at 1 mg/mL in labeling buffer (50 mM Na_2_CO_3_, 100 mM NaCl, pH 9.2) for 1 h. Labeled OMVs were centrifuged at 100,000 ×*g* for 1 h at 4°C, resuspended in PBS (0.2 M NaCl) and washed twice to fully remove the unbound dye. OMVs labeled with R18 (R18-OMVs) were added to HeLa cells pretreated with chlorpromazine (Sigma) at 15 μg/mL or filipin III (Sigma) at 10 μg/mL for 1 h. R18-OMVs were identified using a DMi8 microscope with excitation and emission filter sets at 550 nm and 580 nm, respectively. Alexa Fluor 488-conjugated cholera toxin subunit B (CTB; Thermo Fisher Scientific) was used to label lipid rafts in the plasma membrane at a 1:500 dilution ratio.

In order to observe cytoskeletal reorganization in HeLa cells, cells prepared as described above were stained for actin and nucleus using mouse anti-actin antibody (1:1,000 dilution; Abcam), Alexa Fluor 488-conjugated donkey anti-mouse IgG (1:5,000 dilution; Abcam), and DAPI (1:1,000 dilution; Thermo Fisher Scientific). OMVs were located using rabbit anti-*Salmonella* antibody (1:1,000 dilution) and Alexa Fluor 594-conjugated goat anti-rabbit IgG (1:500 dilution; Abcam). Each of the cover slips was removed from the 24-well culture plate, drained of excess fluid, placed on a glass slide, and 100 μL of BrightMount was added under the glass cover slip (prevent sample drying). After treatment, samples were dried overnight and analyzed using a confocal microscope (SP8 X STED, Leica).

### F-Actin Content Assay

F-actin content in mammalian cells was determined as previously described (McGrath et al., 2000). Briefly, HeLa cells were plated in 24-well culture plates at a density of 2 × 10^5^ cells/well, treated with 50 μL of OMVs fraction (corresponding to approximately 50 μg of protein) and incubated in a CO_2_ incubator at 37°C for 2 h. Actin depolymerization was induced by treating HeLa cells with cytochalasin D at 1 μg/mL for 2 h prior to OMVs addition. Cells treated with DPBS alone were considered as mock-infected cells. Subsequently, cells were washed with DPBS and lysed with lysis buffer (1 M Tris-HCl, pH 7.4, 500 mM EDTA, 5 M NaCl, 10% NP-40, 10% SDS, and 100 × proteinase inhibitor cocktail tablet). Cell lysates were collected and divided into two halves. One-half was centrifuged at 100,000 × *g* for 30 min at 4°C to sediment F-actin. The supernatant was removed leaving 800 μL at the bottom (Higashide et al., 2002). Both, whole cell lysate (the other half) and F-actin fractions were precipitated with 20% TCA. Pellets were suspended in 1 × Laemmli sample buffer, subjected to SDS-PAGE, transferred to PVDF membranes, and probed with mouse anti-actin antibody (1:1,000 dilution; Abcam) and secondary antibody conjugated with horseradish peroxidase (1:700 dilution; Santa Cruz Biotechnology). Immunoblots were developed using West-ZOL Plus western blot detection system. Actin was quantified by densitometric scanning using ChemiDoc MP System.

### Gentamicin Protection Assay

HeLa cells were grown in DMEM supplemented with 10% heat-inactivated FBS and seeded in 24-well culture plates at a density of 2 × 10^5^ cells/well. From an overnight bacterial culture grown in LB broth, 1% (v/v) inoculum was transferred to fresh LB medium and incubated at 37°C with constant shaking for 2.5 h. Monolayered-HeLa cells were pretreated with DMEM or DMEM containing OMVs (50 μg of protein) for 2 h and infected with bacteria for 30 min (in the presence of 5% CO_2_) at a multiplicity of infection (MOI) of 100. After incubation, cells were washed three times with PBS, replenished with fresh DMEM containing gentamicin (100 μg/mL) for 1.5 h, washed three times with PBS, and lysed with 1% Triton X-100 (500 μL/well). To estimate the numbers of bacterial cells adhered to host cells, infected host cells were thoroughly washed using PBS three times and disrupted at 10 min after bacterial addition. Cell lysates were serially diluted and plated on LB agar to determine the numbers of intracellular and adhered bacteria.

### Statistical Analysis

Results were analyzed using Student’s unpaired *t*-test. Data are represented as mean ± standard deviation (SD). A *P-*value <0.05 was considered statistically significant.

## Results

### T3SS1 Effectors Are Secreted via OMVs in *Salmonella*

**FIGURE 1:**
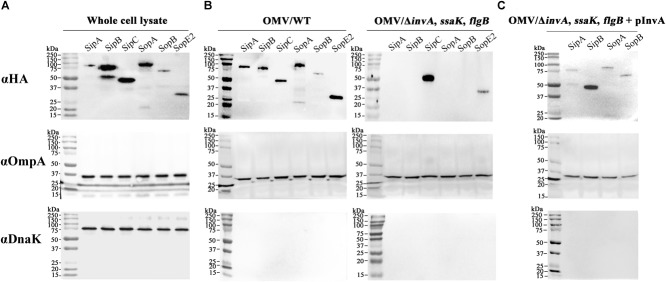
Association of T3SS1 effectors with OMVs released from *Salmonella*. **(A)** Expression of the six *Salmonella* pathogenicity island 1 (SPI-1)-encoded type III secretion system (T3SS1) effectors. Each effector was tagged with hemagglutinin (HA) at its C-terminus and its expression was analyzed using immunoblotting. **(B)** The presence of T3SS1 effectors in the outer membrane vesicles (OMVs) fractions isolated from wild-type (left) or Δ*invA, ssaK, flgB* (right) strains. OMVs were purified from wild-type or Δ*invA, ssaK, flgB* strains expressing each HA-tagged effector and subjected to immunoblot assay. **(C)** Presence of T3SS1 effectors in OMVs fractions isolated from the T3SS1-complemented Δ*invA, ssaK, flgB* strains. The Δ*invA, ssaK, flgB* strains were transformed with pInvA, a plasmid expressing *invA*, and OMVs purified from the T3SS1-complemented Δ*invA, ssaK, flgB* strains were tested to determine the presence of the four T3SS1 effectors (SipA, SipB, SopA, and SopB) in OMVs. The abundant cytosolic protein DnaK was used to detect contamination of OMVs with cytosolic proteins. OmpA, an integral membrane protein of the outer membrane, was used to compare the protein amounts of OMVs between lanes.

Results of our previous study on the proteomic profiling of OMVs in *Salmonella* revealed the presence of effectors and translocator proteins associated with T3SS in OMVs fraction (Bai et al., 2014). Interestingly, T3SS1 effectors were specifically seen in OMVs produced in LB broth, a medium mimicking growth conditions that induced SPI-1 (Ibarra et al., 2010). To validate OMVs-mediated secretion of T3SS1 effectors, six T3SS1 effectors identified from the proteomic profiling of OMVs were each tagged with HA at their C-termini and their presence in the OMVs fraction was examined using immunoblot analysis. All the six T3SS1 effectors were expressed (Figure 1A) and detected in OMVs fractions (Figure 1B) isolated under the LB broth conditions: two T3SS1 translocators of SipB and SipC, and four secretion effectors of SipA, SopA, SopB, and SopE2. Absence of DnaK (a cytosolic protein) in the OMVs fraction excluded the possibility of OMVs contamination with intracellular proteins. The levels of OmpA which is an integral membrane protein abundant in the outer membrane and OMVs were comparable between lanes, indicating equivalent protein amounts between samples.

Outer membrane vesicles-mediated secretion of T3SS1 effectors was re-examined in *Salmonella* lacking T3SSs and flagella to rule out the possibility of accidental incorporation of effectors into extracellular OMVs after their secretion via their cognate secretion system T3SS1. Considering the structural similarity, T3SS2 and flagella were blocked together with T3SS1 to prevent their secretion of T3SS1 effectors. *invA, ssaK*, and *flgB* were deleted to abolish T3SS1, T3SS2, and flagellar apparatus, respectively. InvA forms the export apparatus required for the assembly of functional needle complex of T3SS1 and mutants lacking InvA fail to translocate T3SS1 effectors through their cognate T3SS (Collazo and Galan, 1997; Monjaras Feria et al., 2015). We also observed that T3SS1 effector SopB was not secreted into the culture medium via T3SS1 in Δ*invA* mutant strain (Supplementary Figures S2A, S7). SsaK is a component of T3SS2 C-ring complex associated with the inner membrane and strains with mutations in *ssaK* gene are defective in translocation through T3SS2 (Geddes et al., 2005; Niemann et al., 2011). Accordingly, a Δ*ssaK* mutant strain failed to translocate T3SS2 effector SseJ into the cytosol of macrophages (Supplementary Figure S2B). FlgB constitutes the rod structure of flagellar basal body and its absence impairs the formation of filament and other structures on the membrane surface, thereby blocking flagellum-mediated secretion of proteins (Kubori et al., 1992; Macnab, 2004; Paul et al., 2008). *Salmonella* lacking *flgB* lost its motility, indicating malfunction of flagella (Supplementary Figure S2C). OMVs were purified from the triple mutant strain Δ*invA, ssaK, flgB* and the presence of T3SS1 effectors was examined. Two of the T3SS1 effectors, namely, SipC and SopE2, were detected in OMVs fractions even without functional T3SSs and flagella, indicating their T3SS-independent secretion via OMVs (Figure 1B). We confirmed that the lack of *invA* abolished the secretion of SipC and SopE2 through T3SS1 (Supplementary Figure S3). However, the four secretion effectors including SipA, SipB, SopA, and SopB were absent in OMVs isolated from the triple mutant (Figure 1B). To investigate the possibility of extracellular incorporation of these four effectors into OMVs after T3SS1-mediated secretion, T3SS1 was restored by introducing *invA* in *trans* in the Δ*invA, ssaK, flgB* strain. Consequently, the expression of the four secretion effectors was restored in vesicle fractions purified from the T3SS1-complemented triple mutant strain (Figure 1C).

### T3SS1 Effectors Are Located on the Outer Surface of OMVs

**FIGURE 2:**
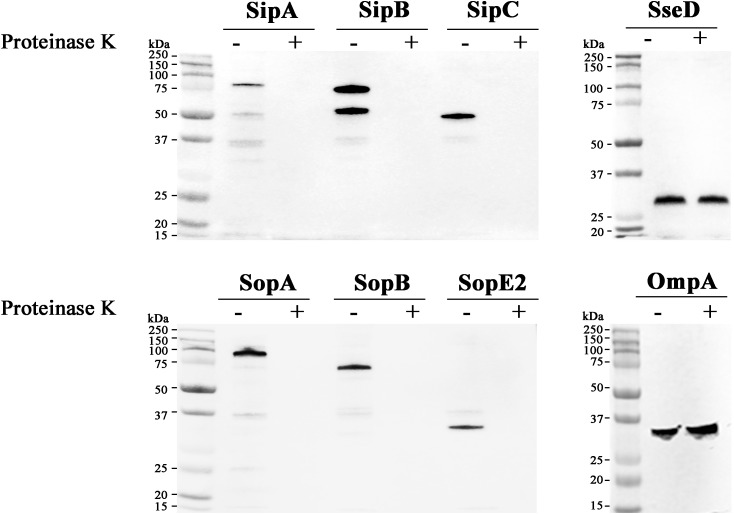
OMVs-associated T3SS1 effectors are located on the outer surface of OMVs. OMVs were isolated from cultures of *Salmonella* strains producing HA-tagged SipA, SipB, SipC, SopA, SopB, SopE2, or SseD. OMVs treated or untreated with proteinase K were precipitated with trichloroacetic acid (TCA) and subsequently analyzed by immunoblotting using anti-HA antibody. SseD protein located in the lumen of vesicles and an integral membrane protein OmpA of OMVs were used as controls.

Our result indicated the possibility of two different mechanisms involved in the association of T3SS1 effectors with OMVs: T3SS-independent incorporation into OMVs and extracellular inclusion in OMVs post T3SS1-mediated secretion. Considering that T3SS1 effectors lack signal sequences accessible to the Sec or TAT pathway, it was intriguing that T3SS1 effectors were accumulated in OMVs without the aid of T3SS1 because vesicle blebs preferentially encapsulate periplasm-derived compounds. To get an insight into the biogenesis of OMVs harboring T3SS1 effectors, the position of T3SS1 effectors in the OMVs compartment was analyzed. OMVs purified from *Salmonella* strains expressing HA-tagged effectors were subjected to proteinase K treatment. OMVs are a substantial compartment composed of phospholipid bilayer that is resistant to a variety of physical and chemical stresses including digestion by proteolytic enzyme (Muralinath et al., 2011). Therefore, the luminal cargoes enclosed within OMVs should be stable against proteinase K treatment, whereas OMVs-associated proteins exposed on the outer surface of vesicles would be susceptible to digestion by proteinase K. Regardless of T3SS-independent incorporation into OMVs or T3SS1-mediated OMVs association, all the six T3SS1 effectors disappeared in the OMVs fractions upon digestion by proteinase K (Figure 2), suggesting their localization on the outer surface of OMVs.

### OMVs-Associated SipA, SipC, and SopE2 Are Translocated Into the Cytoplasm of the Host Cell

**FIGURE 3:**
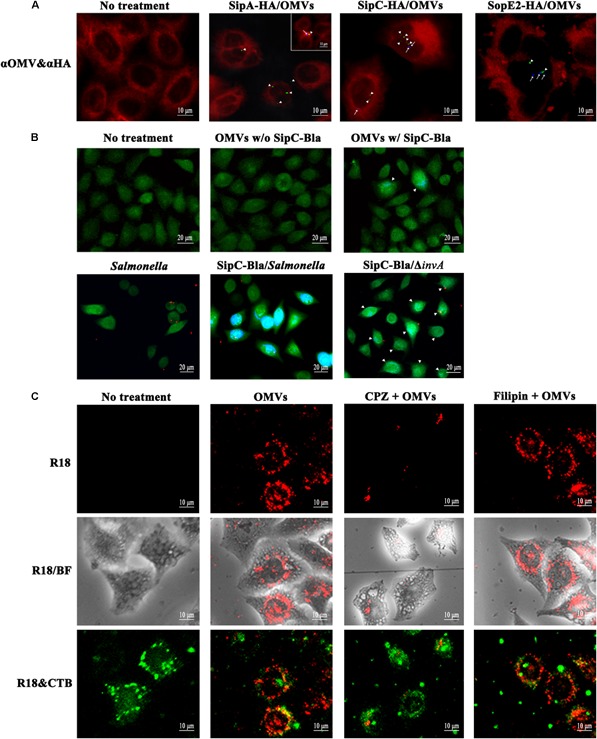
Translocation of SipA, SipC, and SopE2 by OMVs into the cytoplasm of the host cell. **(A)** OMVs-mediated translocation of SipA, SipC, and SopE2 into host cells. HeLa cells were incubated with OMVs harboring SipA-HA, SipC-HA, or SopE2-HA for 2 h. Fixed cells were treated with rabbit anti-*Salmonella* and mouse anti-HA antibodies. Cells were subsequently incubated with Alexa Fluor 405-conjugated anti-rabbit antibody, Alexa Fluor 488-conjugated anti-mouse antibody, and CellMask Deep Red (OMVs in blue; HA-tagged proteins in green; plasma membrane in red). Arrowheads indicate HA-tagged proteins, while arrows indicate OMVs components. The inset shows SipA-HA proximate to a vesicle in the periphery of an epithelial cell. **(B)** CCF4 cleavage assay for SipC-Bla translocation. HeLa cells were treated with OMVs containing SipC-Bla or not for 4 h (upper panel) or *Salmonella* strains (wild-type and Δ*invA*) producing SipC-Bla for 3 h without gentamicin addition (lower panel). SipC-Bla translocated into the cytoplasm of host cells turned the color of CCF4 from green to blue. Cells with localized blue fluorescence are marked with arrow heads. **(C)** Internalization of OMVs through clathrin-mediated endocytosis. OMVs labeled with fluorescence probe R18 (R18-OMVs, red) were added to HeLa cells treated with chlorpromazine (CPZ) or filipin III (Filipin). Lipid rafts in the plasma membrane were labeled with cholera toxin B subunit (CTB) conjugated with Alexa Fluor 488 (green).

Upon contact with intestinal epithelial cells, *Salmonella* uses T3SS1 to translocate a multitude of effector proteins directly into the cytoplasm of the host cell. Our results indicated the incorporation of a few T3SS1 effectors into OMVs and their localization on the outer surface (Figures 1, 2). Many bacterial pathogens utilize OMVs for delivery of active virulence factors into host cells without direct bacteria–host cell contact. Therefore, we speculated whether T3SS1 effectors associated with OMVs could exert their intrinsic activities after OMVs-mediated translocation into host cells. To address the possibility, we first examined whether T3SS1 effectors associated with OMVs could be translocated into the cytoplasm of the host cell without direct interaction between *Salmonella* and the host cell. T3SS1 effectors SipA, SipC and SopE2 mediate bacterial penetration into host epithelial cells through induction of membrane ruffles at the apical surface of the host cell (Clark et al., 1996; Galan, 2001; Lilic et al., 2003; Suárez and Rüssmann, 2010). Epithelial cells were treated with OMVs harboring SipA-HA, SipC-HA, or SopE2-HA and the localization of HA-tagged proteins and OMVs were analyzed using immunofluorescence microscopy. SipA-HA, SipC-HA, and SopE2-HA proteins (arrowheads) were predominantly detected within the cytoplasm of the host cell and OMVs (arrows) intact or disrupted were detected near HA-tagged proteins at 2 h post-addition (Figure 3A). OMVs-mediated SipC translocation into host cells was further verified using CCF4-AM cleavage assay (Figure 3B). SipC protein was tagged with a catalytic domain of β-lactamase (Bla), which can cleave a β-lactam ring of CCF4 and result in blue fluorescence emission when it is translocated into the cytoplasm. HeLa cells treated with OMVs harboring SipC-Bla exhibited blue fluorescence upon the addition of CCF4-AM, whereas epithelial cells untreated or treated with OMVs lacking Bla showed green fluorescence due to FRET of uncleaved CCF4. A *Salmonella* Δ*invA* mutant strain (SipC-Bla/Δ*invA* in Figure 3B) which is defective in T3SS1, thereby secreting SipC only through vesicles (Supplementary Figure S3), also changed the emission spectrum of CCF4 from green to blue when used in HeLa cells infection. These results prove the ability of OMVs in translocating T3SS1 effectors into the cytoplasm of the host cell independent of T3SS1-mediated *Salmonella–*host cell interaction.

To understand how T3SS1 effectors were internalized through OMVs, host cells were treated with chlorpromazine, an inhibitor of clathrin-mediated endocytosis, and filipin III, a cholesterol-binding agent disrupting lipid rafts and caveolae in the plasma membrane. OMVs labeled with fluorescent R18 (R18-OMVs) were internalized into HeLa cells and accumulated perinuclearly at 2 h post-treatment. However, chlorpromazine significantly inhibited the uptake of R18-OMVs, suggesting the possibility of clathrin-mediated endocytosis of OMVs (Figure 3C). Furthermore, OMVs were not colocalized with CTB, a marker labeling lipid rafts in the plasma membrane, ruling out the possibility of OMVs internalization through lipid rafts in the plasma membrane. Uptake of OMVs via clathrin-mediated endocytosis has been observed in *E. coli* (Canas et al., 2016; Bielaszewska et al., 2017). After OMVs internalization into host cells, T3SS1 effectors were likely to separate from OMVs and be directed to their target cellular compartments as evidenced by their dissociation from vesicles at 2 h after OMVs treatments (Figure 3A). These T3SS1 effectors were neither colocalized with LAMP1, a marker protein of late endosome and lysosome, excluding the possibility of their association with lysosomal compartments (Supplementary Figure S4).

### OMVs Harboring T3SS1 Effectors Induce Host Actin Cytoskeletal Rearrangements

**FIGURE 4:**
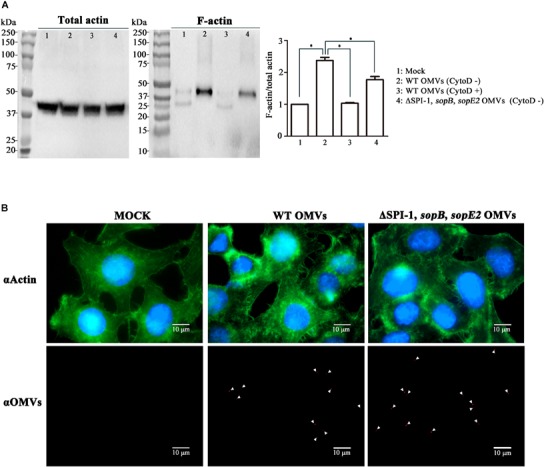
Induction of actin cytoskeletal rearrangements by OMVs harboring T3SS1 effectors in host cell. **(A)** Comparison of F-actin polymerization elicited by treatment with OMVs isolated from wild-type and ΔSPI-1, *sopB, sopE2* strains. Aliquots of cell lysates from equivalent HeLa cells were ultracentrifuged to separate F-actin as described in the Section “Materials and Methods.” F-actin fractions and remnants of total cell lysates were analyzed by immunoblot assay using anti-actin antibody. Cytochalasin D (CytoD) was used to inhibit host cell actin polymerization. The levels of F-actin formation were normalized using the levels of total actin and the ratio of F-actin/total actin in the mock treatment was set to 1.0. Asterisks indicate significant differences with a *P-*value < 0.05. **(B)** Microscopic analysis for cytoskeletal reorganization of HeLa cells induced by OMVs treatments. HeLa cells were treated with OMVs isolated from wild-type and ΔSPI-1, *sopB, sopE2* strains as described above. OMVs, actin, and nucleus are located using anti-*Salmonella* antibody (red), anti-actin antibody (green), and DAPI (blue), respectively, in confocal immunofluorescence microscopy.

We further investigated whether T3SS1 effectors translocated by OMVs could exert their intrinsic activities in the host cell. Once their translocation via the cognate secretion system T3SSs, the effector proteins of *Salmonella* trigger a complex set of signaling events in the host cell. This leads to modulation of host cellular functions facilitating bacterial colonization and proliferation in the host. *Salmonella*-induced membrane ruffling in intestinal epithelial cells is accomplished by coordinated action of the four T3SS1 effectors associated with OMVs; SopB and SopE2 induce rearrangement of the actin cytoskeleton, SipA and SipC stabilize the rearranged architecture of actin bundles (Clark et al., 1996; Galan, 2001; Suárez and Rüssmann, 2010). Activation of Rho GTPases RhoG and Cdc42 by SopB and SopE2 leads to the formation of highly branched actin cytoskeleton (Stender et al., 2000; Zhou et al., 2001). SipA and SipC bind directly to actin and cooperate with each other to stimulate the formation of actin filaments (Zhou et al., 1999; Myeni and Zhou, 2010). Actin rearrangement drives the formation of membrane ruffles that protrude outward and engulf *Salmonella.* We therefore explored the T3SSs-independent ability of OMVs in inducing cytoskeletal reorganization in the host cell. OMVs were harvested from wild-type *Salmonella* and a mutant strain devoid of *sopB, sopE2*, and the SPI-1 loci (referred to as ΔSPI-1, *sopB, sopE2*). Considering the locations of *sipA* and *sipC* genes in SPI-1, the ΔSPI-1, *sopB, sopE2* strain was supposed to produce OMVs lacking the four T3SS1 effectors SipA, SipC, SopB, and SopE2. The abundance of actin bundles was compared in epithelial cells treated with OMVs. Immunoblot assay revealed enhanced formation of F-actin by OMVs derived from the wild-type strain than those derived from the ΔSPI-1, *sopB, sopE2* strain (Figure 4A). Treatment of HeLa cells with cytochalasin D disrupting actin polymerization nullified the influence of wild-type OMVs on activating F-actin formation (Figure 4A). Fluorescence microscopic observation accordantly revealed increased actin rearrangements in epithelial cells by wild-type OMVs than that caused by OMVs lacking the four T3SS1 effectors (Figure 4B). HeLa cells treated with wild-type OMVs showed actins condensed and bundled. Although OMVs lacking the four T3SS1 effectors also caused actin rearrangements, the intense actin accumulation was not detectable. The addition of wild-type OMVs did not activate Cdc42 but increased its production in HeLa cells in comparison with untreated cells (Supplementary Figure S5). Interestingly, OMVs lacking the four T3SS1 effectors reduced the activation level of Cdc42. Activated Cdc42 not only facilitates actin cytoskeleton remodeling but also stimulates the induction of proinflammatory cytokines such as IL-8 production (Chen et al., 1996). Taken together, these results suggest that T3SS1 effectors translocated to the cytoplasm by OMVs induced rearrangement of actin cytoskeleton in the host cells independent of direct *Salmonella–*host cell interaction.

### OMVs Harboring T3SS1 Effectors Promote the Invasive Ability of *Salmonella*

**FIGURE 5:**
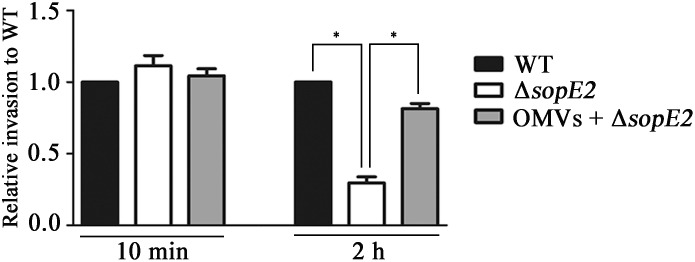
OMVs complemented the attenuated invasion of Δ*sopE2* strain into host cell. Confluent monolayers of HeLa cells were pretreated with Dulbecco’s modified Eagle’s medium (DMEM) or DMEM containing wild-type OMVs for 2 h and subsequently infected with wild-type or Δ*sopE2* *Salmonella* strains. Extracellular bacteria were removed by gentamicin treatment and intracellular bacteria were enumerated at 2 h post-infection. Bacterial cells adhered onto host cells were counted at 10 min post infection without gentamicin treatment. The bars represent relative invasion abilities normalized using the levels of wild-type *Salmonella* from five independent experiments. Asterisks indicate significant differences for a *P-*value < 0.05.

The observation that OMVs harboring T3SS1 effectors triggered actin filamentation in the absence of cellular interaction between *Salmonella* and the host cell led us to investigate further the role of OMVs in promoting the invasive ability of *Salmonella*. A mutant strain lacking *sopE2* showed adhesion ability comparable to wild-type *Salmonella* when added to HeLa cells but was attenuated in invasion into host cells by approximately threefold in comparison with wild-type *Salmonella*, probably due to the compromised membrane ruffling in the host cells. However, addition of wild-type OMVs to HeLa cells prior to infection with Δ*sopE2* strain restored the attenuated invasion to almost 80% of wild-type invasion (Figure 5). These results suggest the exertion of biological activity by OMVs harboring active T3SS1 effectors during *Salmonella* infection in contributing to the invasive ability of the bacteria.

## Discussion

*Salmonella* is a leading cause of bacterial infection worldwide. Among a myriad of virulence factors acquired by horizontal gene transfer during its evolution into a pathogen, SPIs are the best-studied virulence determinants. In *Salmonella*, SPI-1 and SPI-2 are discrete genetic loci primarily associated with invasion into the host cell and intracellular survival, respectively (Srikanth et al., 2011). SPIs possess a multitude of genes encoding virulence effectors, their regulators and chaperons, and a needle-like apparatus for their translocation into the cytosol of host cells. To date, more than 30 virulence factors have been reported to be secreted by a specialized secretion apparatus known as T3SS in *Salmonella*. Although SPI T3SS is considered as the primary secretion system, recent studies suggest the possibility of an alternative secretion mechanism responsible for the secretion of virulence factors in *Salmonella*. Niemann et al. (2011) identified six T3SS effectors and two additional proteins that were translocated into the host cytoplasm independent of T3SS. Yoon et al. (2011) identified a couple of PagK-homologous proteins required for *Salmonella* virulence, which were produced under SPI-2 inducing conditions but were translocated via OMVs independent of T3SS2 injectisome.

Most Gram-negative bacteria constitutively produce OMVs. OMVs produced by pathogenic bacteria contain multiple virulence factors such as adhesins, toxins, and immunomodulatory compounds. They directly mediate bacterial adhesion to host cell, invasion into host cells, and manipulation of host immune responses (Schwechheimer and Kuehn, 2015). In the present study, the possibility of employing OMVs as an alternative delivery vehicle for T3SS1 effectors was examined using multiple experimental approaches. HA-tagged six T3SS1 effectors (SipA, SipB, SipC, SopA, SopB, and SopE2) were found to be secreted through OMVs *in vitro* (Figure 1A). SipC and SopE2 were associated with OMVs without the aid of the three secretion systems T3SS1, T3SS2, and flagellar apparatus (Figure 1B). This result confirms T3SSs-independent OMVs-mediated secretion of T3SS1 effectors. The four T3SS1 effectors (SipA, SipB, SopA, and SopB) were detected in wild-type OMVs but not in OMVs produced from Δ*invA, ssaK, flgB* (Figure 1B). This could be explained based on the likelihood that the bacterial culture conditions used in our study might not precisely represent the *in vivo* environment where these effector proteins are produced and secreted for *Salmonella* virulence. Alternatively, the volume of bacterial culture used for isolating OMVs might have been insufficient to determine their association with OMVs in immunoblot analysis. Interestingly, defects in T3SSs and flagella decreased the mRNA levels of SPI-1 regulators and six of the T3SS1 effectors, resulting in decreased protein levels of effectors SipA and SipB (Supplementary Figures S6, S8). Therefore, SipA and SipB might not be detectable in OMVs produced from Δ*invA, ssaK, flgB* because of their low levels, although their mRNAs were expressed along with *sipC* in the same operon.

T3SS effectors are thought to be directed to their cognate secretion apparatus by signal sequences presumably located at their N-termini or 5’-UTR and are delivered directly to the cytoplasm of the host cell across the two bacterial membranes and the host plasma membrane (Sorg et al., 2005). However, they usually lack a conserved signal sequence except for a small numbers of effectors belonging to *Salmonella* translocated effector (STE) family (Miao and Miller, 2000). Instead, various chaperon proteins recognize their respective T3SS substrates through distinctive N-terminal amino acids of effector proteins and coordinate their timely translocation into the cytosol of the host cell (Buttner, 2012). Therefore, it was not surprising that the six T3SS1 effectors of this study did not possess signal peptides for passing through the Sec or the TAT system in the inner membrane, as predicted by *in silico* computational analysis (data not shown). However, considering that OMVs preferentially enclose periplasm-derived compounds, it is intriguing that T3SS1 effectors lacking signal sequences accessible to the Sec or TAT pathway were associated with OMVs without the aid of T3SSs. Dual export strategies, namely, OMVs-associated and OMVs-independent secretion have been reported to be functional in the translocation of several toxins and virulence factors in different pathogenic bacteria (Bonnington and Kuehn, 2014). Despite being a sole substrate for type IV secretion system (T4SS), CagA is associated with the surface of OMVs in *H. pylori* (Olofsson et al., 2010). CagA delivered by OMVs to host cells was colocalized with the tight junction protein ZO-1 and induced the binding of histone H1 to ATP, suggesting a different endocytic route other than T4SS for CagA delivery into gastric epithelial cells (Backert and Tegtmeyer, 2017). Considering the structural and functional similarity between T3SS and T4SS, their effectors might hijack OMVs in a similar way in diverse bacterial pathogens. Vesicle blebs occurring in the vicinity of T3SS1 injectisome might incorporate the T3SS1 substrate drifting along the apparatus (Bonnington and Kuehn, 2014). Taking into consideration the availability of insufficient information related to the structural determinants for OMVs cargo, an unidentified complex tag conserved in three-dimensional structure present in T3SS1 effectors might direct their T3SS1-independent association with OMVs.

With regard to the mechanisms of OMVs internalization into non-phagocytic cells, several pathways have been suggested (Rewatkar et al., 2015; O’Donoghue and Krachler, 2016): macropinocytosis, clathrin-mediated endocytosis, caveolin-mediated endocytosis, non-caveolin lipid raft-mediated endocytosis, and lipid raft-mediated membrane fusion. To get an insight into OMVs biogenesis at the contact with HeLa cells, OMVs labeled with fluorescent R18 were added to HeLa cells which were pre-treated with chlorpromazine or filipin III (Figure 3C). Treatment with chlorpromazine significantly reduced OMVs entry into HeLa cells, suggesting that OMVs might be internalized via clathrin-mediated endocytosis. Many studies have demonstrated a role for clathrin in OMVs internalization. Diverse virulence factors associated with OMVs, including shiga toxin, cholera toxin, and vacuolating toxin, exploit clathrin-mediated endocytosis to gain entry into host cells (Sandvig and van Deurs, 2002; Parker et al., 2010; Bielaszewska et al., 2017). In the case of *E. coli* OMVs, virulence factors (Stx2a and CdtV-B) associated with OMVs rapidly separated from the vesicles and moved to their target compartments, while OMVs were gradually colocalized with early endosomes, late endosomes, and lysosomes in a time-dependent manner after internalization through endocytosis (Bielaszewska et al., 2017). We also observed that vesicles of intact or disrupted forms were occasionally colocalized with LAMP1, a marker protein of late endosome and lysosome, at 4 h post-treatments, while an T3SS1 effector SipC was rarely associated with vesicles or LAMP1 at the same time (Supplementary Figure S4).

During the infection process, *Salmonella* can fine-tune the production and secretion of effectors in response to the environmental conditions. *Salmonella*, while crossing the intestinal mucus layer, recognizes the micro-environmental cues of high osmolarity, low oxygen tension, and basic pH in the layer of epithelial cells and accordingly turns on the expression of SPI-1 genes for translocation of effectors into epithelial cells using T3SS1 injectisome (Gong et al., 2010; Srikanth et al., 2011). Once internalized, *Salmonella* resides inside a vacuole named *Salmonella-*containing vacuole (SCV) and experiences drastic environmental changes, including nutrient starvation, low pH, and low concentrations of divalent cations (Ca^2+^ and Mg^2+^) and phosphates (Deiwick et al., 2006). These stimuli induce the production of T3SS2 effectors in *Salmonella* and their translocation into the cytoplasm of the host cell with the aid of T3SS2 (Srikanth et al., 2011). In contrast to the intrinsic T3SSs-mediated translocation of effectors requiring intercellular *Salmonella–*host cell contact, alternative OMVs-mediated delivery of T3SS effectors might provide additional benefit to *Salmonella.* OMVs pinching off sustainedly from the bacterial surface are able to deliver virulence factors independent of timely and spatially controlled T3SSs. In addition, the small size of OMVs enables them to diffuse across a variety of host membranous structures and the bilayered vesicle structure protects their cargo from hostile extracellular stresses (Schwechheimer and Kuehn, 2015). In this context, T3SS effectors associated with OMVs might be delivered to multiple host cells independent of *Salmonella–*host cell contact and promote structural and physiological changes in the host cell that are beneficial for *Salmonella* in compromising the host defense mechanism. Because of the versatile features of OMVs, *Salmonella* might exploit OMVs as an alternative delivery vehicle for T3SS effectors that functions in a manner distinct from the tightly controlled T3SSs.

## Data Availability

All relevant data is contained within the manuscript and the supplementary files. The raw data supporting the conclusions of this manuscript will be made available on request.

## Author Contributions

SK, SIK, EK, and HY conceived, designed, and coordinated the study. SIK, SK, EK, and SH performed the experiments and interpreted the data. SK and HY wrote the manuscript. All authors took part in discussing the results and in reviewing the manuscript.

## Conflict of Interest Statement

The authors declare that the research was conducted in the absence of any commercial or financial relationships that could be construed as a potential conflict of interest.
