# Corrigendum: Secretion of *Salmonella* Pathogenicity Island 1-Encoded Type III Secretion System Effectors by Outer Membrane Vesicles in *Salmonella Enterica* Serovar Typhimurium

**DOI:** 10.3389/fmicb.2019.00411

**Published:** 2019-03-12

**Authors:** Seul I Kim, Seongok Kim, Eunsuk Kim, Seo Yeon Hwang, Hyunjin Yoon

**Affiliations:** ^1^Department of Molecular Science and Technology, Ajou University, Suwon, South Korea; ^2^Department of Applied Chemistry and Biological Engineering, Ajou University, Suwon, South Korea

**Keywords:** *Salmonella*, outer membrane vesicles (OMVs), *Salmonella* pathogenicity island 1 (SPI-1), type III secretion system (T3SS), virulence, effector

In the original article, there was a mistake in Table S2 as published. Some primers listed in Table S2 had incorrect sequences. The corrected Table S2 appears below.

**Table S2:** Primers used in this study.

**Primers**	**DNA sequence from 5^**′**^to 3^**′**^**
Red-*sipA*-HA-F	TCGGGTTATTACTACCGTTGATGGCTTGCAC ATGCAGCGTTATCCGTATGATGTTCCTGATTATGC TTAATGTAGGCTGGAGCTGCTTCG
Red-*sipA*-HA-R	TTGCTTCAATATCCATATTCATCGCAT CTTTCCCGGTTAAATTCCGGGGATCCGTCGACC
*sipA*-D-F	CTATGTGTTAAGTAATGTGCTGG
*sipA*-D-R	AAATCCAATGAGTCAGCGTAAAG
Red-*sipB*-2HA-F	TGCGGATGCTTCGCGTTTTATTCTGCGCCA GAGTCGCGCAATTCCGGGGATCCGTCGACC
Red-*sipB*-2HA-R	TAATTAACATATTTTTCTCCCTT TATTTTGGCAGTTTTTAGTGTAGGCTGGAGCTGCTCC
*sipB*-D-F	CAGATTCAGCAGTGGCTTAAAC
*sipB*-D-R	ACTGACTTTACTGCTGCTAATAC
Red-*sipC*-2HA-F	AGCATCCGCACTCGCTGCTATCGCAGGCAATA TTCGCGCTATTCCGGGGATCCGTCGACC
Red-*sipC*-2HA-R	AATCACACCCATGATGGCGTATAGATGACCTTTCAGATTAGT GTAGGCTGGAGCTGCTCC
*sipC*-D-F	AATTAGCCAGGTGAATAACCGG
*sipC*-D-R	ATGTTCTGTGGTAGACGGTAC
Red-*sopA*-2HA- F	CCCGAGTGTTCTGTCATCCATCCTGCCACTGGCCTGGGCG ATTCCGGGGATCCGTCGACC
Red-*sopA*-2HA-R	CGCTGTGTCCCTTAATTCCATGCGGGTTGAGGCTGGACTAGT GTAGGCTGGAGCTGCTCC
*sopA*-D-F	ATATTCCCATCCAGCGAACAG
*sopA*-D-R	GTCCATCTGAAATTACTGATTCTG
Red-*sopB*-2HA-F	TTGGCAGTCAGTAAAAGGCATTTCTTCATT AATCACATCTATTCCGGGGATCCGTCGACC
Red-*sopB*-2HA-R	ACGATTTAATAGACTTT CCATATAGTTACCTCAAGACTCAGT GTAGGCTGGAGCTGCTCC
*sopB*-D-F	AATAGCGGTAACCTGGAGATTC
*sopB*-D-R	ATTTCCAGTGTATGATCGGATTC
Red-*sopE2*-2HA-F	TATAGAAAATATTGCGAATAAGTATCTTCAGAA TGCCTCCATTCCGGGGATCCGTCGACC
Red-*sopE2*-2HA-R	ATTCATATGGTTAATAG CAGTATTGTATTTACTACCATCAGT GTAGGCTGGAGCTGCTCC
*sopE2*-D-F	CATAATCAGCAGGTATCTTTTAAAG
*sopE2*-D-R	CTGGTTGTGCAGGTATTTAGAG
Red-ΔSPI1-F	GCATAACGGCATTGTTATCGAATCGCTC ATAAAGCGTTCAGTGTAGGCTGGAGCTGCTTC
Red-ΔSPI1-R	CAGCCAACGGTGATATGGCCTTATAAGG CTTGCAGTCTTTATTCCGGGGATCCGTCGACC
ΔSPI1-D-F	CTGCTATTCAGGAAACATAC
ΔSPI1-D-R	AAATAAAAAAAGCAGCAGCG
pBAD30-*invA*-F	GAACAGCGTCGACCTATTGAA
pBAD30-*invA*-R	CCAAATGTTGCATAAAGCTTTTC
pBAD30-D-F	CCATAAGATTAGCGGATCC
pBAD30-D-R	CAGGCAAATTCTGTTTTATC
Red-SipC-Bla-F	GCATCCGCACTCGCTGCTATCGCA GGCAATATTCGCGCTCTGTCTCTTATACACATCTCA
Red-SipC-Bla-R	TCACACCCATGATGGCGTATAGAT GACCTTTCAGATTACTGTCTCTTATACACATCTGGT
SipC-Bla-D-F	GCCACAGTATTAAAAACGTG
SipC-Bla-D-R	TTGAGATCCAGTTCGATGTAACC
*sipA*-qRT-F	TGGCTGCCAGAAACAAAGAA
*sipA*-qRT-R	AAGATTGCTGCGGGTTAACG
*sipB*-qRT-F	CAGCGAAGGGCAATTGACAT
*sipB*-qRT-R	ACTCAATCATCGCCTGCCATA
*sipC*-qRT-F	AGGCTGATAGCAAACTGTCTGGTA
*sipC*-qRT-R	ACTCAATCATCGCCTGCCATA
*sopA*-qRT-F	TACGTCACAAAGCCAACCTCTCT
*sopA*-qRT-R	GTGGCATTTGCAGCCAGATA
*sopB*-qRT-F	GCTCGCCCGGAAATTATTGT
*sopB*-qRT-R	TAGAGGTTATGCAGCGAGTGGTT
*sopE2*-qRT-F	GAAAAAACAACGGAGAAGGACATT
*sopE2*-qRT-R	TGCAGGCTAAAACGATCTGACA
*hilA*-qRT-F	GCTGCACCAGGAAAGCATTAA G
*hilA*-qRT-R	CGAAGTCCGGGAATACATCTGA
*hilC*-qRT-F	GCCGCTGAAGAGGTGAGTTTTA
*hilC*-qRT-R	AATATTTCCAGCCCCCATACG
*hilD*-qRT-F	GCTGTTCCTGCTTACTGCTTTTC
*hilD*-qRT-R	AATGTTGTAAACGCGCTCCTTT
*invF*-qRT-F	GCGGAAAAGCGAAGAGTGAA
*invF*-qRT-R	AACGGCTAATTGGGTGATGTTC
*gyrB*-qRT-F	TCGCTCAGCAGTTCGTTCAT
*gyrB*-qRT-R	GATTGCGGTGGTTTCCGTAA

The authors apologize for this error and state that this does not change the scientific conclusions of the article in any way. The original article has been updated.

