# Supplementary material for: Secretion of Salmonella Pathogenicity Island 1-Encoded Type III Secretion System Effectors by Outer Membrane Vesicles in Salmonella enterica Serovar Typhimurium
Source: Front Microbiol. 2018 Nov 23;9:2810. doi: 10.3389/fmicb.2018.02810 (PMC6266720; doi:10.3389/fmicb.2018.02810)
Supplement: Supplementary file 1 [file Data_Sheet_1.pdf]

Supplementary information

Secretion of *Salmonella* Pathogenicity Island 1-Encoded Type III Secretion System Effectors by Outer Membrane Vesicles in *Salmonella enterica* serovar Typhimurium

Seul I Kim[1], Seongok Kim[1†], Eunsuk Kim[1], Seo Yeon Hwang[1], and Hyunjin Yoon[1,2,*]

† These authors have contributed equally to this work

*** Correspondence:** Hyunjin Yoon: yoonh@ajou.ac.kr

Materials and Methods

Transmission electron microscopy (TEM)

OMVs were purified as described in the material and methods section. To condensate OMVs, the vesicles were centrifuged using an Amicon Ultra-4 centrifugal filter (100 kDa MWCO, Millipore, USA). OMVs suspended in PBS were mounted onto 200-mesh Formvar/Carbon-coated copper grids (Woomyoung Inc., South Korea) for 10 min. The grids were washed two times with ultrapure water and stained with 2% uranyl acetate for 1 min. Images of OMVs were obtained using a Talos L120C transmission electron microscope (Thermo Fisher Scientific, USA).

cAMP assay

RAW264.7 macrophage cells were grown in DMEM supplemented with 10% heat-inactivated FBS and seeded in 24-well culture plates at a density of 5 × 10^5 cells/well. Bacteria producing CyaA′-tagged SseJ were grown overnight in LB broth and 1% (v/v) inoculum was transferred to fresh LB medium. The cells were incubated with constant shaking at 37°C for 9 h. Monolayered RAW264.7 cells were infected with bacteria at a MOI of 100 for 30 min and replenished with fresh DMEM containing gentamicin (100 μg/mL) for 1.5 h to remove extracellular bacteria. After further incubation in fresh DMEM containing gentamicin at 20 μg/mL for 7 h, cells were lysed with 0.1 M HCl for 10 min and the supernatant was used for cAMP assays according to the manufacturer's instructions (Direct cAMP ELISA kit; Enzo life sciences, USA). Absorbance at 405 nm was measured using a colorimetric 96-well plate reader and the levels are expressed as pg/mL.

Motility assay

Wild-type *Salmonella* and Δ*flgB* strains were cultured overnight in LB broth at 37°C and 1% (v/v) inoculum was transferred to fresh LB medium and incubated for 2.5 h until mid-log phase. Bacterial cultures were diluted to obtain an OD_{600} of 1.0. Aliquots (2 μL) of each strain were injected into 0.3% soft-agar plates and incubated at 37°C for 6 h.

Cdc42 activation assay

HeLa cells were seeded in T25 flask at a density of 5 × 10^5 cells/flask, treated with OMVs (50 μg of protein) or DPBS, and incubated at 37°C and 5% CO_2 for 2 h. Cdc42 activation was measured using a Cdc42 Activation Assay Kit (Cytoskeleton Inc., USA) according to the manufacturer's

instructions. Briefly, cells were washed once with ice-cold PBS, lysed in cell lysis buffer (25 mM Tris pH 7.5, 10 mM $MgCl_2$, 0.5 mM NaCl, and 2 % Igepal) supplemented with 1 × protease inhibitor cocktail, and centrifuged at 10,000 × *g* for 1 min at 4°C. Cell lysates were quantified using Bio-Rad protein assay (Bio-Rad, USA). Cell lysates containing equivalent amounts of protein (approximately 200 μg) were incubated with 10 μg of GST-PAK-PBD beads at 4°C with gentle end-over-end rotation (18 rpm) for 1 h. Active Cdc42 was pulled down by centrifugation at 5,000 × *g* at 4°C for 3 min. Cell lysates mixed with GDP (negative control) or GTPγS (positive control) were used as respective controls. Beads were washed once in washing buffer (25mM Tris pH 7.5, 30 mM $MgCl_2$, 40 mM NaCl). Protein samples on beads were dissolved in 2 × Laemmli sample buffer, and analyzed by SDS-PAGE and immunoblot. Cdc42 and GAPDH were detected using anti-Cdc42 (1:250 dilution; Cytoskeleton Inc.) and anti-GAPDH (1:2,000 Dilution; Abcam, UK) antibodies in combination with secondary antibody conjugated with horseradish peroxidase (1:700 dilution; Santa Cruz Biotechnology, USA). GAPDH was used in parallel for normalizing protein loading between the lanes. The bands were visualized using EZ-Western Lumi Pico western blot detection kit (DAEILLAB SERVICE, South Korea) and analyzed by ChemiDoc MP System.

qRT-PCR analysis

Salmonella cells at log phase in LB broth medium were treated with RNAprotect bacteria reagent (Qiagen, USA). Total RNA was isolated using RNeasy mini kit (Qiagen). Isolated RNA samples were treated with RNase-free DNase (Ambion) to remove residual DNA. cDNA was synthesized using RNA to cDNA EcoDry Premix (Takara, Japan). Target mRNA was quantified using StepOne Plus real-time PCR system (Applied Biosystems, USA). mRNA levels of each gene

were normalized relative to mRNA levels of the house keeping gene *gyrB*. Primers used in qRT-PCR are listed in Table S2.

Supplementary Tables

Table S1. Bacterial strains and plasmids used in this study

Strains and plasmids	Genotype	References or sources
***Salmonella* Typhimurium strain**		
14028	wild-type *S. enterica* serovar Typhimurium	ATCC 14028
SK01	*sipA*-HA	This study
SK02	*sipC*-HA	This study
SK03	*sipB*-HA	This study
SK04	*sopB*-HA	This study
SK05	*sopE2*-HA	This study
SK06	*sopA*-HA	This study
SK07	*sipA*::*kan*-HA	This study
SK08	*sipC*::*kan*-HA	This study
SK09	*sipB*:: *kan*-HA	This study
SK10	*sopB*:: *kan*-HA	This study
SK11	*sopE2*::*kan*-HA	This study
SK12	*sopA*::*kan*-HA	This study
YC0398	*ssaK*::*kan*	(Yoon et al., 2011)
YC0595	*invA*::*cm*	(Yoon et al., 2011)

SK13	*sopB*-HA harboring pBAD30	This study
SK14	*sopB*-HA/Δ*invA* harboring pBAD30	This study
SK15	*sopB*-HA/Δ*invA* harboring pInvA	This study
YC0594	*flgB*::*cm*	(Yoon et al., 2011)
SK16	Δ*flgB*	This study
SK17	*sipA*-HA, Δ*invA*, *ssaK*, *flgB*	This study
SK18	*sipC*-HA, Δ*invA*, *ssaK*, *flgB*	This study
SK19	*sipB*-HA, Δ*invA*, *ssaK*, *flgB*	This study
SK20	*sopB*-HA, Δ*invA*, *ssaK*, *flgB*	This study
SK21	*sopE2*-HA, Δ*invA*, *ssaK*, *flgB*	This study
SK22	*sopA*-HA, Δ*invA*, *ssaK*, *flgB*	This study
SK23	*sipA*-HA, Δ*invA*, *ssaK*, *flgB* harboring pInvA	This study
SK24	*sipB*-HA, Δ*invA*, *ssaK*, *flgB* harboring pInvA	This study
SK25	*sopA*-HA, Δ*invA*, *ssaK*, *flgB* harboring pInvA	This study
SK26	*sopB*-HA, Δ*invA*, *ssaK*, *flgB* harboring pInvA	This study
YC0638	*sseJ-cyaA'*	(Bai et al., 2014)
YC0582	*sseJ-cyaA'*, Δ*ssaK*	(Yoon et al., 2011)
SK27	Δ*sipC*	This study
SK28	Δ*sopE2*	This study
SK29	Δ*invA*	This study

SY01	SPI-1::*kan*	This study
SY02	ΔSPI-1, *sopE2*, *sopB*	This study
EK0251	*sipC-bla*	This study
EK0259	*sipC-bla* harboring pQE30::mCherry	This study
EK0271	*sipC-bla*, Δ*invA* harboring pQE30::mCherry	This study
**Plasmid**		
pKD13	Amp^R, FRT Kan^R FRT, PS1 PS4, *ori*R6K$_\Psi$	(Datsenko and Wanner, 2000)
pKD13-2HA	pKD13 derivative harboring HA-tag	(Ansong et al., 2009)
pKD46	Amp^R, P_{BAD}, *gam-beta-exo*, *ori*R101	(Datsenko and Wanner, 2000)
pCP20	rep_{pSC101}^{ts}, Amp^R, Cm^R, cl857 λ P_R *flp*	(Datsenko and Wanner, 2000)
pBAD30	Amp^R, *araC*, P_{BAD}, pACYC184 *ori*,	(Guzman et al., 1995)
pInvA	pBAD30 harboring *invA*	This study
pMini-Tn5-BLAM	pMini-Tn5-cycler harboring β-lactamase	(Yoon et al., 2011)
pQE30::mCherry	pQE30 harboring mCherry	(Kim et al., 2018)

Table S2. Primers used in this study

primers	DNA sequence from 5' to 3'
Red-*sipA*-HA-F	TCGGGTTATTACTACCGTTGATGGCTTGCACATGCAGCGTTATCCGTATGATGTTCCTGATTATGCTTAATGTAGGCTGGAGCTGCTTCG
Red-*sipA*-HA-R	TTGCTTCAATATCCATATTCATCGCATCTTTCCCGGTTAAATTCCGGGGATCCGTCGACC
sipA-D-F	CTATGTGTTAAGTAATGTGCTGG
sipA-D-R	AAATCCAATGAGTCAGCGTAAAG
Red-*sipB*-2HA-F	TGCGGATGCTTCGCGTTTTATTCTGCGCCAGAGTCGCGCAATTCCGGGGATCCGTCGACC
Red-*sipB*-2HA-R	TAATTAACATATTTTTCTCCCTTTATTTTGGCAGTTTTTAGTGTAGGCTGGAGCTGCTCC
sipB-D-F	CAGATTCAGCAGTGGCTTAAAC
sipB-D-R	ACTGACTTTACTGCTGCTAATAC
Red-*sipC*-2HA-F	AGCATCCGCACTCGCTGCTATCGCAGGCAATATTCGCGCTATTCCGGGGATCCGTCGACC
Red-*sipC*-2HA-R	AATCACACCCATGATGGCGTATAGATGACCTTTCAGATTAGTGTAGGCTGGAGCTGCTCC

sipC-D-F	AATTAGCCAGGTGAATAACCGG
sipC-D-R	ATGTTCTGTGGTAGACGGTAC
Red-*sopA*-2HA- F	CCCGAGTGTTCTGTCATCCATCCTGCCACTGGCCTGGGCGATTC CGGGGATCCGTCGACC
Red-*sopA*-2HA-R	CGCTGTGTCCCTTAATTCCATGCGGGTTGAGGCTGGACTAGT GTAGGCTGGAGCTGCTCC
sopA-D-F	ATATTCCCATCCAGCGAACAG
sopA-D-R	GTCCATCTGAAATTACTGATTCTG
Red-*sopB*-2HA-F	TTGGCAGTCAGTAAAAGGCATTTCTTCATTAATCACATCTATTC CGGGGATCCGTCGACC
Red-*sopB*-2HA-R	ACGATTTAATAGACTTTCCATATAGTTACCTCAAGACTCAGT GTAGGCTGGAGCTGCTCC
sopB-D-F	AATAGCGGTAACCTGGAGATTC
sopB-D-R	ATTTCCAGTGTATGATCGGATTC
Red-*sopE2*-2HA-F	TATAGAAAATATTGCGAATAAGTATCTTCAGAATGCCTCCATT CCGGGGATCCGTCGACC
Red-*sopE2*-2HA-R	ATTCATATGGTTAATAGCAGTATTGTATTTACTACCATCAGT GTAGGCTGGAGCTGCTCC

sopE2-D-F	CATAATCAGCAGGTATCTTTTAAAG
sopE2-D-R	CTGGTTGTGCAGGTATTTAGAG
Red-ΔSPI1-F	GCATAACGGCATTGTTATCGAATCGCTCATAAAGCGTTCAGTG TAGGCTGGAGCTGCTTC
Red-ΔSPI1-R	CAGCCAACGGTGATATGGCCTTATAAGGCTTGCAGTCTTTATT CCGGGGATCCGTCGACC
ΔSPI1-D-F	CTGCTATTCAGGAAACATAC
ΔSPI1-D-R	AAATAAAAAAAGCAGCAGCG
pBAD30-*invA*-F	GAACAGCGTCGACCTATTGAA
pBAD30-*invA*-R	CCAAATGTTGCATAAAGCTTTTC
pBAD30-D-F	CCATAAGATTAGCGGATCC
pBAD30-D-R	CAGGCAAATTCTGTTTTATC
Red-SipC-Bla-F	GCATCCGCACTCGCTGCTATCGCAGGCAATATTCGCGCTCTGT CTCTTATACACATCTCA
Red-SipC-Bla-R	TCACACCCATGATGGCGTATAGATGACCTTTCAGATTACTGTCT CTTATACACATCTGGT
SipC-Bla-D-F	GCCACAGTATTAAAAACGTG

SipC-Bla-D-R	TTGAGATCCAGTTCGATGTAACC
sipA-qRT-F	TGGCTGCCAGAAACAAAGAA
sipA-qRT-R	AAGATTGCTGCGGGTTAACG
sipB-qRT-F	CAGCGAAGGGCAATTGACAT
sipB-qRT-R	ACTCAATCATCGCCTGCCATA
sipC-qRT-F	AGGCTGATAGCAAACTGTCTGGTA
sipC-qRT-R	ACTCAATCATCGCCTGCCATA
sopA-qRT-F	TACGTCACAAAGCCAACCTCTCT
sopA-qRT-R	GTGGCATTTGCAGCCAGATA
sopB-qRT-F	GCTCGCCCGGAAATTATTGT
sopB-qRT-R	TAGAGGTTATGCAGCGAGTGGTT
sopE2-qRT-F	GAAAAAACAACGGAGAAGGACATT
sopE2-qRT-R	TGCAGGCTAAAACGATCTGACA
hilA-qRT-F	GCTGCACCAGGAAAGCATTAA G
hilA-qRT-R	CGAAGTCCGGGAATACATCTGA

hilC-qRT-F	GCCGCTGAAGAGGTGAGTTTTA
hilC-qRT-R	AATATTTCCAGCCCCCATACG
hilD-qRT-F	GCTGTTCCTGCTTACTGCTTTTC
hilD-qRT-R	AATGTTGTAAACGCGCTCCTTT
invF-qRT-F	GCGGAAAAGCGAAGAGTGAA
invF-qRT-R	AACGGCTAATTGGGTGATGTTC
gyrB-qRT-F	TCGCTCAGCAGTTCGTTCAT
gyrB-qRT-R	GATTGCGGTGGTTTCCGTAA

2

Supplementary Figures

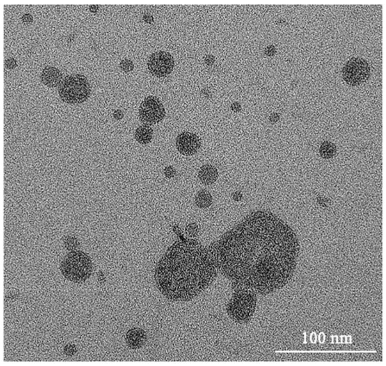

Supplementary Figure S1. TEM image of purified OMVs.

Outer membrane vesicles (OMVs) were isolated as described in materials and methods. Purified OMVs were placed onto a formvar/carbon-coated 200-mesh copper grid. OMVs were stained with 2% uranyl acetate and observed using TEM.

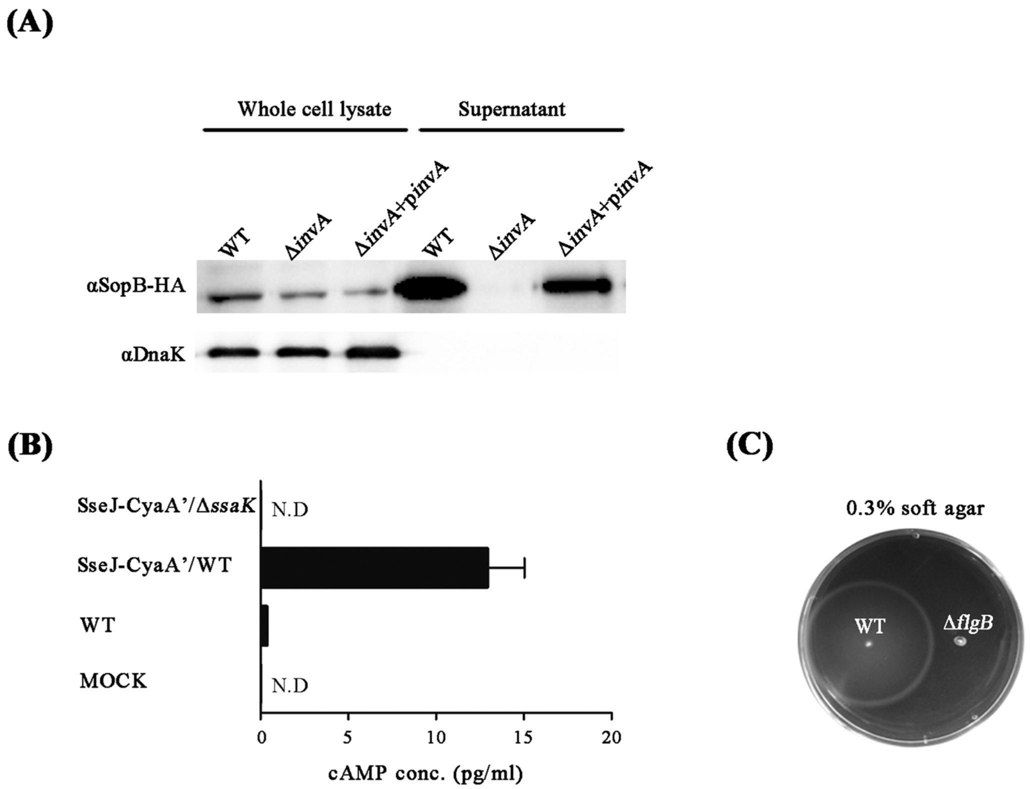

Supplementary Figure S2. Phenotypic analysis of *Salmonella* mutant strains lacking *invA*, *ssaK* or *flgB*.

(A) Inhibition of SopB secretion in the absence of *invA*. Whole cell lysates and supernatant fractions filtered using a 100-kDa-pore-sized membrane to remove OMVs were prepared from *Salmonella* strains cultured in LB broth and subjected to immunoblot analysis using anti-HA and anti-DnaK antibodies. **(B)** Inhibition of translocation of SseJ in the absence of *ssaK*. Macrophages were infected with *Salmonella* strains producing CyaA′-tagged SseJ and the intracellular cAMP levels were measured at 9 h post-infection to compare the translocation of SseJ-CyaA′ between wild-type and Δ*ssaK* strains. cAMP assay was performed in triplicate. N.D: not detected. **(C)** Absence of motility caused by the lack of *flgB*. *Salmonella* cells including

wild-type and Δ*flgB* stains were injected into semi-solid agar plates and incubated for 6 h. Full-length blot images are represented in Supplementary Figure S7.

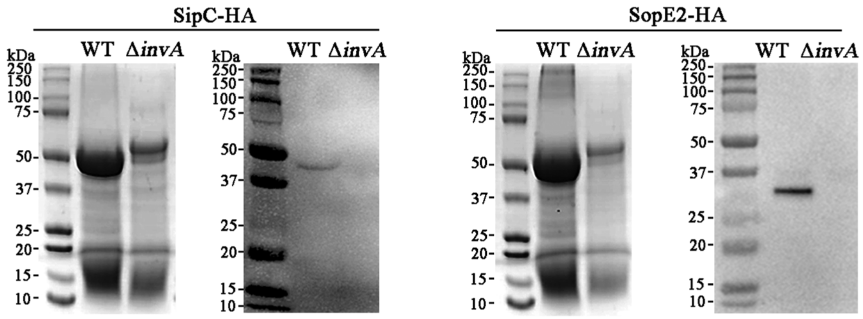

Supplementary Figure S3. Blocked secretion of SipC and SopE2 through T3SS1 in the absence of *invA*.

Wild-type and Δ*invA* mutant strains producing SipC-HA and SopE2-HA were cultivated in LB broth conditions for 4 h and the culture supernatants were filtered to remove bacterial cells. The filtrates were re-filtered using a 100-kDa-pore-sized membrane to remove OMVs and subjected to TCA precipitation. The supernatant fractions devoid of vesicles were analyzed using SDS-PAGE (left) and immunoblotting (right) for each T3SS1 effector. HA-tagged proteins were identified using anti-HA and anti-DnaK antibodies.

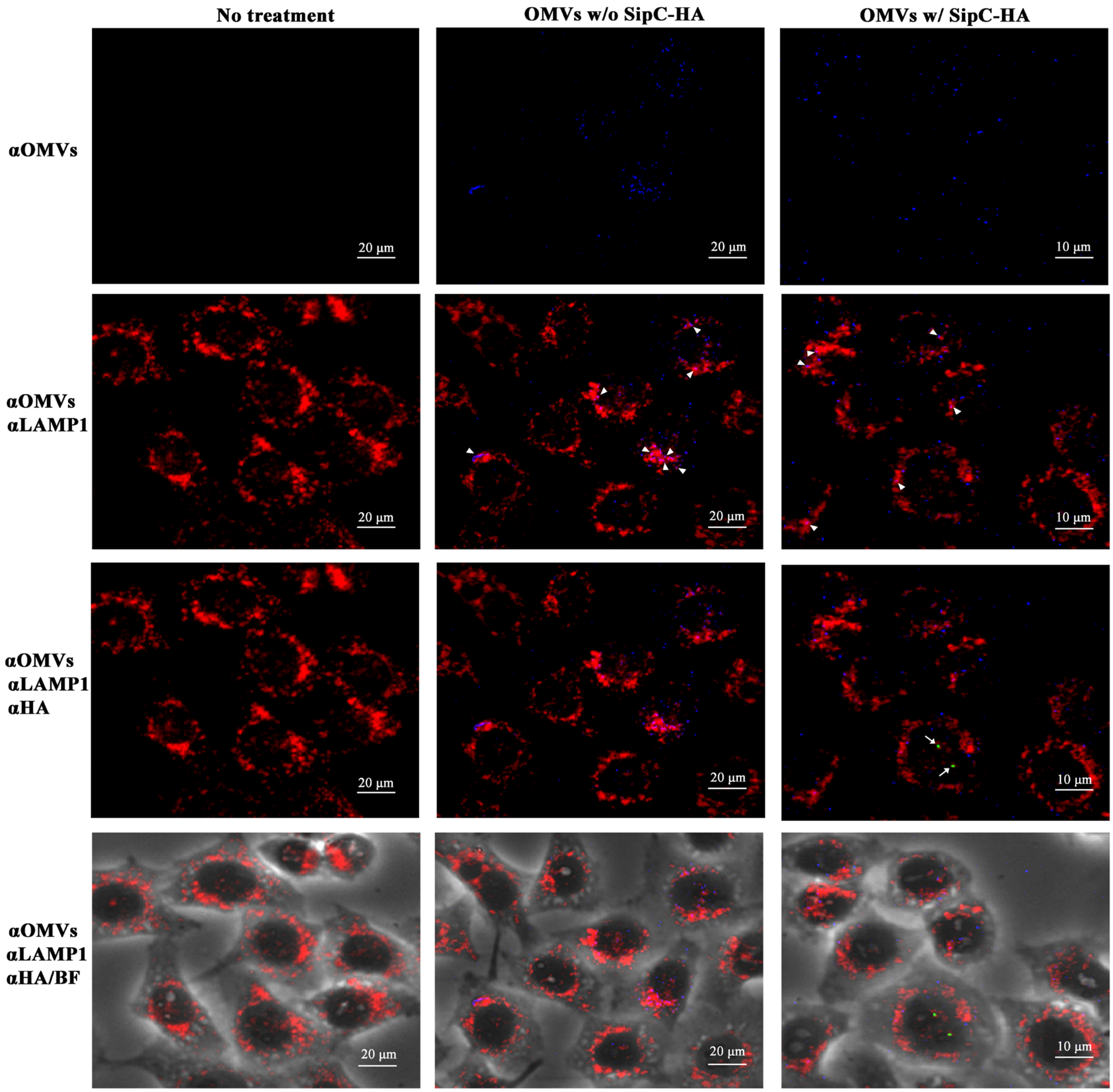

Supplementary Figure S4. Localization of OMVs, SipC, and LAMP1 inside HeLa cells at 4 h post-treatments.

HeLa cells were treated with OMVs containing SipC-HA or not for 4 h. OMVs (blue) were labeled with mouse anti-*Salmonella* Typhimurium LPS and Alexa Fluor 350-conjugated goat anti-mouse antibodies. SipC-HA (green) was detected using chicken anti-HA and Alexa Fluor 488-conjugated goat anti-chicken antibodies. LAMP1 protein (red) was indentified using rabbit

anti-LAMP1 and Alexa Fluor 647-conjugated donkey anti-rabbit antibodies. Colocalization of intact or disrupted OMVs with LAMP1 is indicated with arrow heads, while SipC-HA distinct from LAMP1 is marked with arrows.

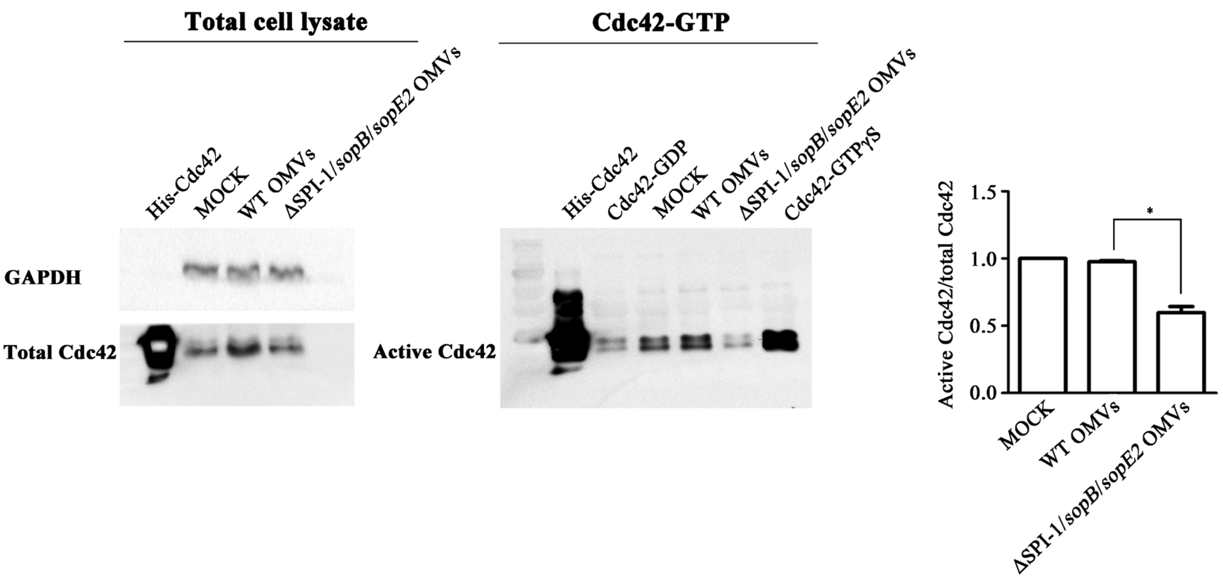

Supplementary Figure S5. Analysis of Cdc42 activation in OMVs-treated host cells.

HeLa cells were treated with OMVs isolated from wild-type and ΔSPI-1, *sopB*, *sopE2* strains for 2 h. Comparison of Cdc42 activation induced by OMVs isolated from wild-type and ΔSPI-1, *sopB*, *sopE2* strains. Aliquots of cell lysates containing equivalent protein amounts were treated with GST-PAK-PBD beads to pull down Cdc42-GTP (active Cdc42) as described in materials and methods. Total lysates and pulled down active Cdc42 fractions were subjected to immunoblotting using anti-Cdc42 antibody. Cell lysates mixed with GDP (Cdc42-GDP) or GTPγS (Cdc42- GTPγS) were used as negative or positive controls, respectively. Total lysates were further probed with anti-GAPDH antibody to normalize the amounts of proteins between samples. The proportion of active Cdc42 to total Cdc42 was graphically shown. The ratio of the mock treatment was set to 1.0. An asterisk indicates a significant difference with a *P*-value < 0.05.

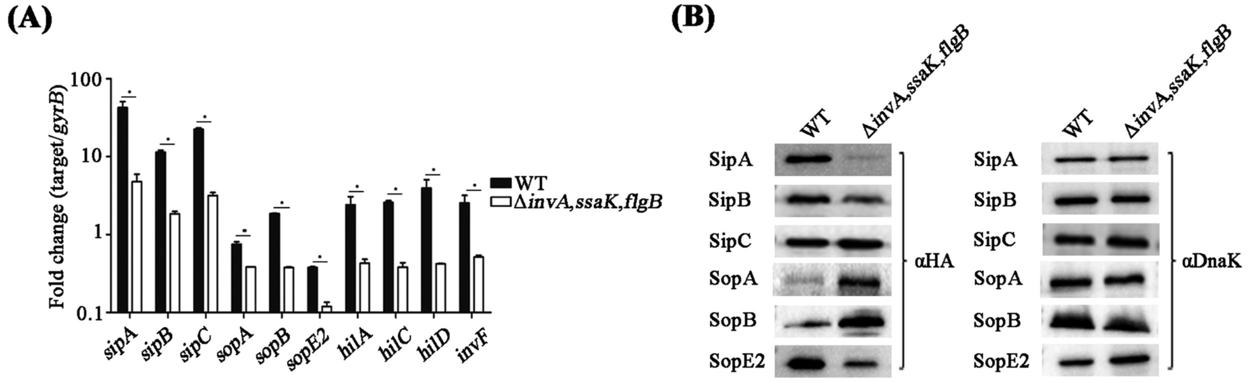

Supplementary Figure S6. Comparison of transcriptional and translational expression of T3SS1 effectors and regulators between wild-type and Δ*invA*, *ssaK*, *flgB* strains.

(A) Real-time polymerase chain reaction **(**qRT-PCR) analysis of T3SS1-associated genes encoding effectors and regulators. Transcription level of each gene was normalized to that of *gyrB* in each strain and plotted relatively. The experiments were performed in triplicate. Asterisks indicate significant differences for a *P*-value < 0.05. **(B)** Protein expression of the six T3SS1 effectors in wild-type and Δ*invA*, *ssaK*, *flgB* strains. Equivalent protein amounts of each strain were subjected to immunoblot analysis. Full-length blot images are represented in Supplementary Figure S8.

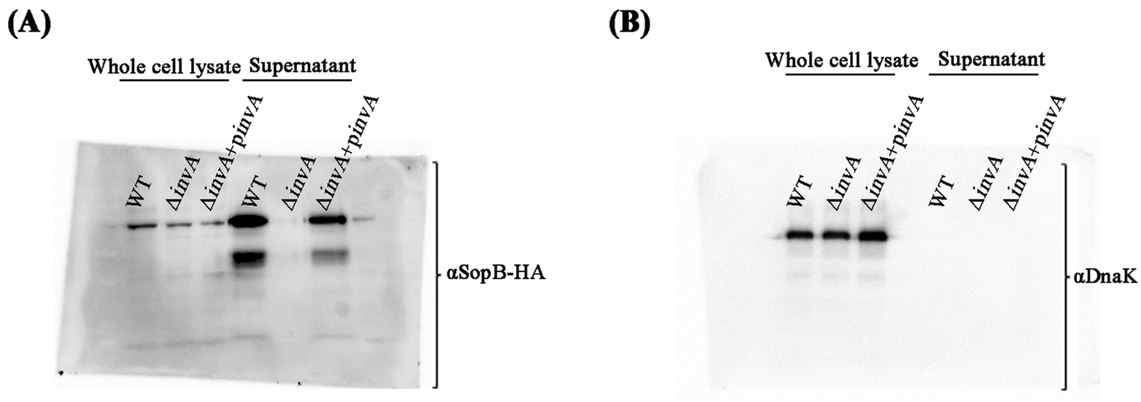

Supplementary Figure S7. Full-images of Figure S2A.

(A) A blot image of SopB secretion in the absence of *invA*. **(B)** A blot image of DnaK expression. Whole cell lysates and supernatant fractions filtered using a 100-kDa-pore-sized membrane were subjected to immublot analysis. SopB-HA and DnaK proteins were identified with anti-HA and anti-DnaK antibodies, respectively.

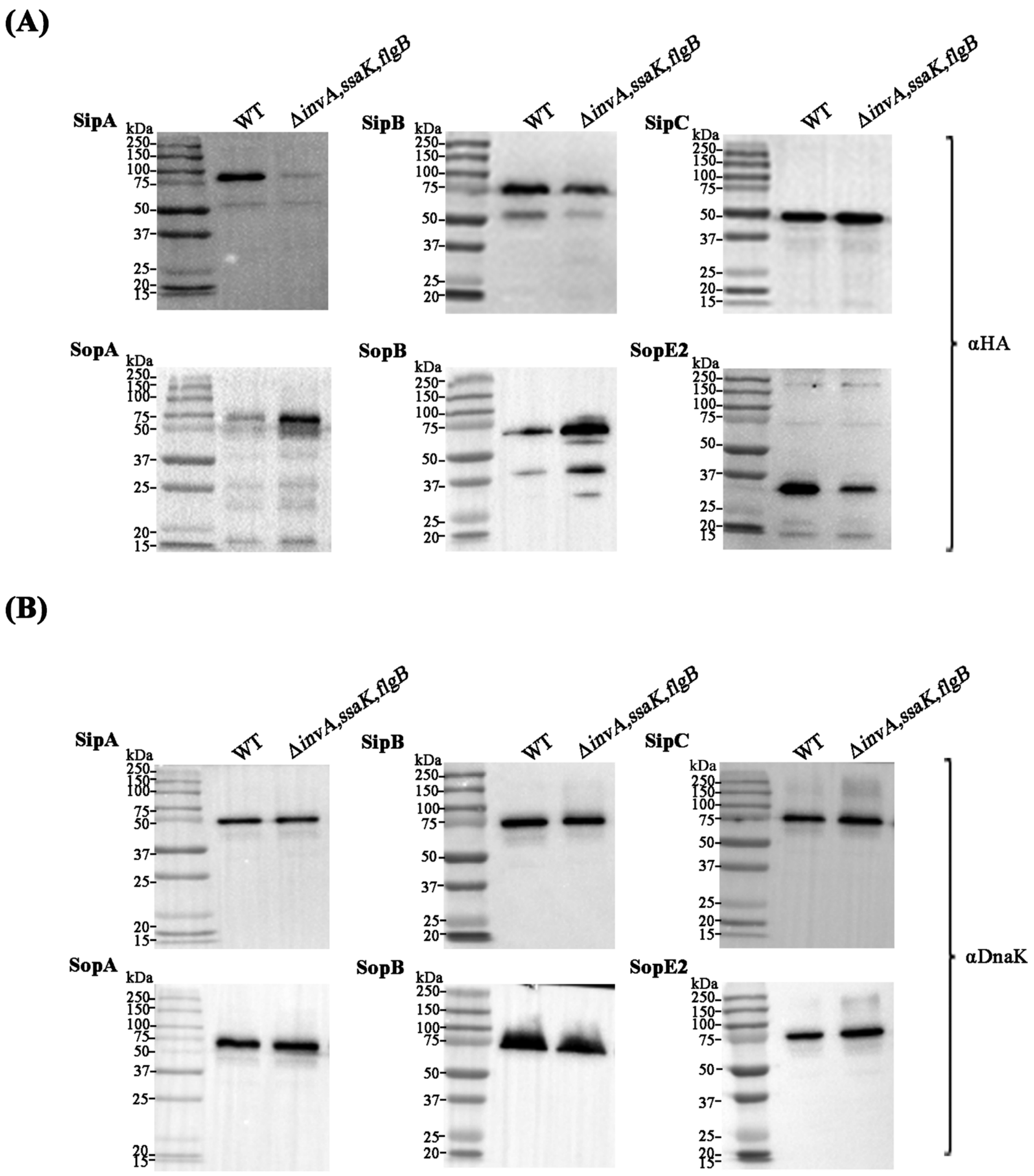

Supplementary Figure S8. Full-images of Figure S6B**.**

(A) Comparison of protein expression of the six T3SS1 effectors between wild-type and Δ*invA*, *ssaK*, *flgB* strains. **(B)** Detection of DnaK between wild-type and Δ*invA*, *ssaK*, *flgB* strains. Whole cell lysates were subjected to SDS-PAGE, and bands were transferred to PVDF membrane and probed with anti-HA and anti-DnaK antibodies, respectively.

References

Ansong, C., Yoon, H., Porwollik, S., Mottaz-Brewer, H., Petritis, B.O., Jaitly, N., Adkins, J.N., Mcclelland, M., Heffron, F., and Smith, R.D. (2009). Global systems-level analysis of Hfq and SmpB deletion mutants in *Salmonella*: implications for virulence and global protein translation. *PLoS One* 4**,** e4809.

Bai, J., Kim, S.I., Ryu, S., and Yoon, H. (2014). Identification and characterization of outer membrane vesicle-associated proteins in *Salmonella enterica* serovar Typhimurium. *Infect Immun* 82**,** 4001-4010.

Datsenko, K.A., and Wanner, B.L. (2000). One-step inactivation of chromosomal genes in *Escherichia coli* K-12 using PCR products. *Proc Natl Acad Sci U S A* 97**,** 6640-6645.

Guzman, L.M., Belin, D., Carson, M.J., and Beckwith, J. (1995). Tight regulation, modulation, and high-level expression by vectors containing the arabinose PBAD promoter. *J Bacteriol* 177**,** 4121-4130.

Kim, S., Yoon, H., and Ryu, S. (2018). New virulence factor CSK29544_02616 as LpxA binding partner in Cronobacter sakazakii. *Sci Rep* 8**,** 835.

Yoon, H., Ansong, C., Adkins, J.N., and Heffron, F. (2011). Discovery of *Salmonella* virulence factors translocated via outer membrane vesicles to murine macrophages. *Infect Immun* 79**,** 2182-2192.
